# Astrocytic Vesicle Mobility in Health and Disease

**DOI:** 10.3390/ijms140611238

**Published:** 2013-05-27

**Authors:** Maja Potokar, Nina Vardjan, Matjaž Stenovec, Mateja Gabrijel, Saša Trkov, Jernej Jorgačevski, Marko Kreft, Robert Zorec

**Affiliations:** 1 Laboratory of Neuroendocrinology-Molecular Cell Physiology, Institute of Pathophysiology, Faculty of Medicine, University of Ljubljana, Zaloška 4, 1000 Ljubljana, Slovenia; E-Mails: maja.potokar@mf.uni-lj.si (M.P.); nina.vardjan@mf.uni-lj.si (N.V.); matjaz.stenovec@mf.uni-lj.si (M.S.); mateja.gabrijel@mf.uni-lj.si (M.G.); sasa.trkov@mf.uni-lj.si (S.T.); jernej.jorgacevski@mf.uni-lj.si (J.J.); marko.kreft@mf.uni-lj.si (M.K.); 2 Celica Biomedical Center, Tehnološki park 24, 1000 Ljubljana, Slovenia; 3 Biotechnical Faculty, University of Ljubljana, Večna pot 111, 1000 Ljubljana, Slovenia

**Keywords:** astrocyte, glia, vesicle, trafficking, gliotransmitter, antigen presentation, neuroinflammation, amyotrophic lateral sclerosis, multiple sclerosis

## Abstract

Astrocytes are no longer considered subservient to neurons, and are, instead, now understood to play an active role in brain signaling. The intercellular communication of astrocytes with neurons and other non-neuronal cells involves the exchange of molecules by exocytotic and endocytotic processes through the trafficking of intracellular vesicles. Recent studies of single vesicle mobility in astrocytes have prompted new views of how astrocytes contribute to information processing in nervous tissue. Here, we review the trafficking of several types of membrane-bound vesicles that are specifically involved in the processes of (i) intercellular communication by gliotransmitters (glutamate, adenosine 5′-triphosphate, atrial natriuretic peptide), (ii) plasma membrane exchange of transporters and receptors (EAAT2, MHC-II), and (iii) the involvement of vesicle mobility carrying aquaporins (AQP4) in water homeostasis. The properties of vesicle traffic in astrocytes are discussed in respect to networking with neighboring cells in physiologic and pathologic conditions, such as amyotrophic lateral sclerosis, multiple sclerosis, and states in which astrocytes contribute to neuroinflammatory conditions.

## 1. Introduction

Astrocytes support and integrate many functions in the central nervous system (CNS). The list of their indispensable roles in brain tissue has grown rapidly in the past two decades and includes: regulation of synaptogenesis, neuronal transmission, brain microcirculation, formation/maintenance of the blood–brain barrier, formation/resolution of brain edema, metabolic support to neurons, and pathologic immune response [1–12]. Intercellular communication between astrocytes and surrounding tissue is supported by several mechanisms: through channels, transporters, and the exchange of molecules by exocytotic and endocytotic processes [13–18]. By the latter two processes, signaling molecules are released from, or are internalized into, membrane-bound vesicles and reach their cytoplasmic destination by trafficking, which involves interaction with the cytoskeleton [19,20].

Membrane-bound vesicles in astrocytes carry several molecules, such as amino acids, nucleotides, peptides, transporters, water channels, and receptors [13,21–30]. Their efficient delivery to the target destination in the cell is governed by vesicle mobility. It has been established recently that cytoplasmic vesicle mobility over distances of several micrometers is a tightly regulated process, involving cell signaling pathways originating at the cell membrane or in the cell interior. The vesicle mobility pattern is changed also by alterations in the dynamics of cytoskeletal filaments or the activity of cytoskeletal regulatory molecules. Under pathologic conditions (ischemia, brain injury, brain edema, brain inflammation), different triggers alter vesicle mobility, as shown by several model studies in recent years [20,21,31–36].

Intense research in the field of single vesicle trafficking in astrocytes has provided much new data and new perspectives on the role of astrocytes in brain functioning. This review focuses on the mobility properties of exocytotic vesicles that transport various gliotransmitters (glutamate, adenosine 5′-triphosphate [ATP], atrial natriuretic peptide [ANP], and brain-derived neurotrophic factor [BDNF]), transporters (excitatory amino acid transporter 2 [EAAT2]), water channels (aquaporins [AQP4]), and antigen-presenting receptors (major histocompatibility complex class [MHC-II]), and their role in health and disease. Although electron microscopy studies have revealed that astrocytes contain clear- and dense-core vesicles [13], it is currently unknown whether the trafficking of these are distinct, therefore the aim here is to review studies where vesicle mobility was monitored in real-time in astrocytes.

## 2. Gliotransmitter-Loaded Secretory Vesicles

Gliotransmitters are chemicals released from glial cells and are synthesized by and/or stored in glia [13]. Storage of gliotransmitters in membrane-bound vesicles in astrocytes has been reported for the amino acids glutamate and d-serine [15,23,25,30,37–39], for ATP [22,26] and for the peptides ANP [15,27], BDNF [40], and tissue plasminogen activator (tPA) [41]. Several vesicle types have been studied systematically in vesicle mobility experiments performed on primary cultured astrocytes to explore their responsiveness in mobility to different physiologic and pathologic stimuli at the single vesicle level.

**Figure 1:**
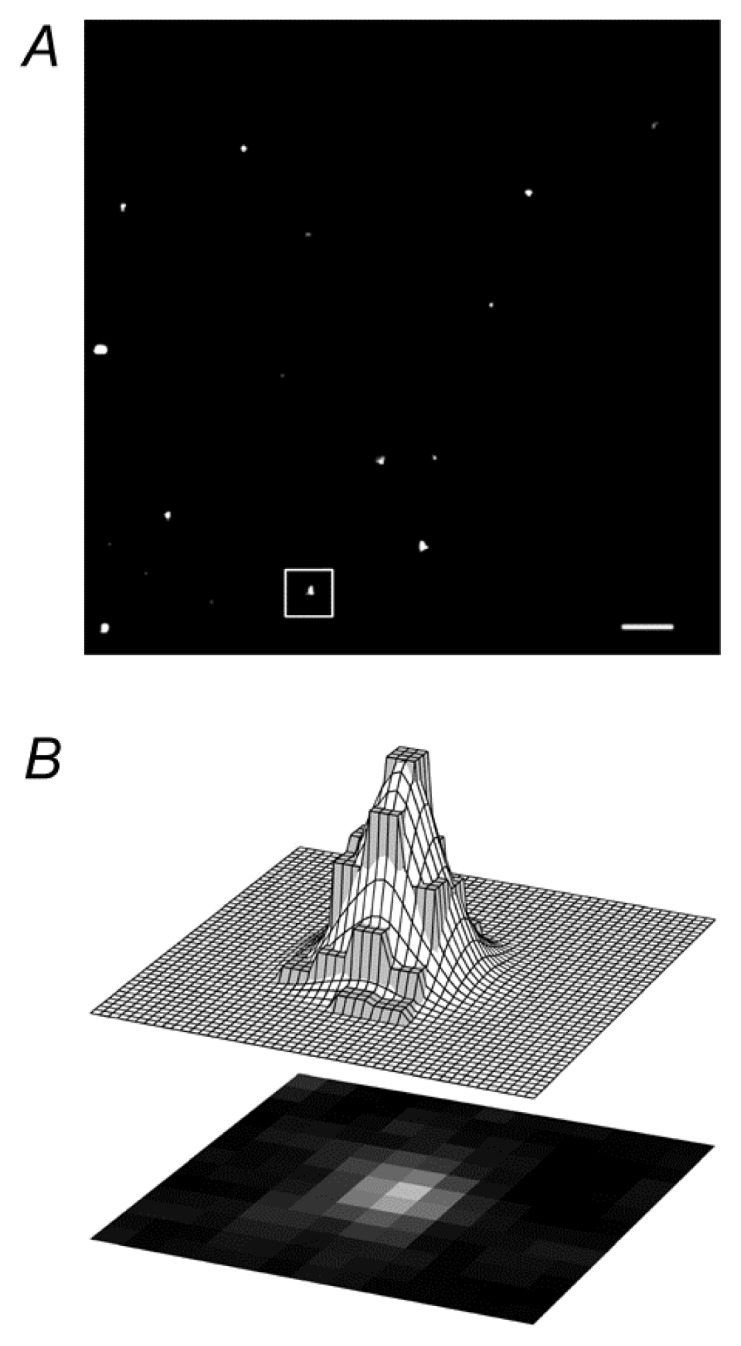

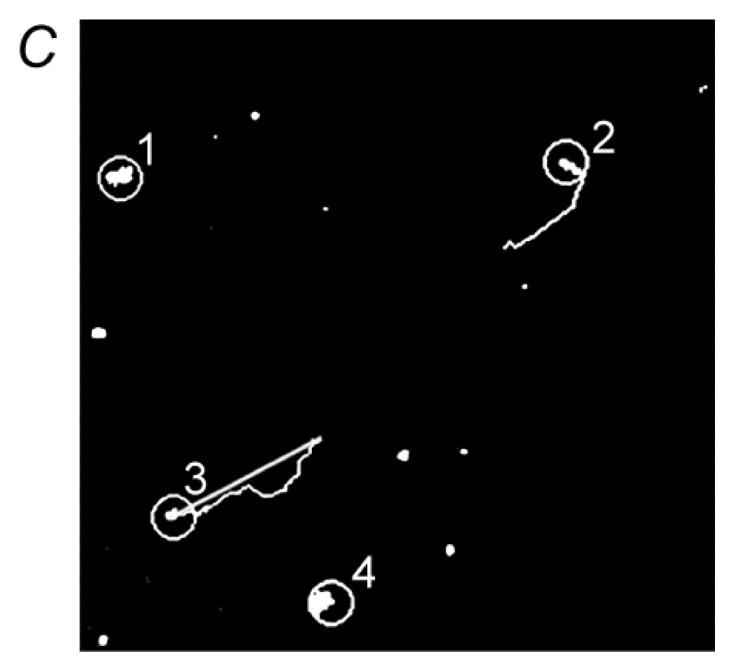
Distinct types of mobility of pro-ANP-Emd labeled vesicles. (**A**) Magnification of part of a rat astrocyte with fluorescently labeled vesicles. Bar: 2.5 μm; (**B**) An example of a two-dimensional Gaussian curve fitted to a single vesicle [see area outlined in (**A**)]. Lower panel shows a single vesicle as nine bright pixels. Upper panel presents a schematic view of the two-dimensional Gaussian curve fitted to the pixel intensity distribution representing a vesicle. The pixel intensities are gray and the surface of the two-dimensional Gaussian curve is white; (**C**) Vesicles analyzed with ParticleTR software; (**C**) Examples of two modes of vesicle mobility. Vesicles 2 and 3 show directional mobility, whereas vesicles 1 and 4 have nondirectional mobility. The line on vesicle 3 depicts the maximal displacement that the vesicle attained in the observation time of 15 s. Reproduced with permission from [19].

**Figure 2:**
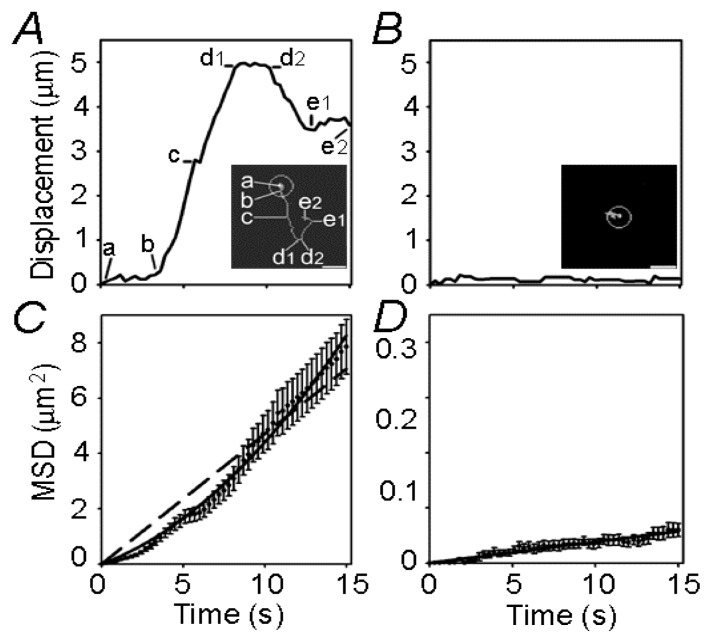
Single vesicle mobility tracks. (**A**) Displacement from the origin versus time for a directional vesicle. The first quarter of the tracking vesicle’s mobility from the origin consists of almost constant displacements (a to b) and during this time the vesicle remains close to the origin of tracking [see inset (**A**)]. In the next third of the tracking time (b–d1), vesicle displacements increase rapidly in a preferential direction with a brief pause (c). After a short period with equal displacements (d1–d2), the vesicle seems to move backwards, however, apparently not on the same track (d2–e1 and inset); (**B**) The displacement from the origin for a nondirectional vesicle. Minor mobility was observed and the vesicle did not translocate far from the origin of tracking (see inset). The mean square displacement (MSD) shown in (**C**,**D**) was calculated according to the equation MSD = [d(*t*) − d(*t* + Δ*t*)]^2^; (**C**) The MSD of directional vesicles. The dashed line represents a linear function fitted to the data using an equation with the form MSD (μm^2^) = (0.4702 ± 0.0099) × time (s). The upwardly curving line represents a quadratic function fitted to the data following the equation MSD (μm^2^) = (0.2189 ± 0.0148) × time (s) + (0.0221 ± 0.0013) × time^2^ (s^2^); (**D**) The MSD of nondirectional vesicles. The linear function was fitted to the data following the equation MSD (μm^2^) = (0.0038 ± 0.0001) × time (s). Bar: 2.5 μm. Reproduced with permission from [19].

Spontaneous mobility of membrane-bound vesicles in astrocytes was first described by Potokar *et al*. in 2005 [19], and subsequently confirmed by Crippa *et al*. in 2006 [42]. Several parameters can be estimated to characterize vesicle mobility. Briefly, vesicle mobility can be determined by the total track length (TL) that a vesicle travels in a given time, the average velocity, the displacement and the directionality index (consisting of the ratio of maximal displacement/total track length (Figure 1). Maximal displacement (MD) was determined as a measure of the maximal net translocation of vesicles during the observation [43]. In astrocytes, two distinct types of vesicle mobility have been described, consistent with other cell types [19,44–46]. These were described as directional (vesicle tracks displaying a straight line) and nondirectional (vesicle tracks displaying a contorted line), and different modes of vesicle mobility were characterized by switching the mobility between the two modes of mobility [19] (Figure 2).

### 2.1. Amino Acid-Loaded Vesicles

In astrocytes, glutamate is packaged into vesicles by the vesicular glutamate transporters (VGLUTs) VGLUT1, VGLUT2 and VGLUT3 [13,47]. Although the existence of VGLUT1 in mouse astrocytes was questioned [48], VGLUT1-containing vesicles in rat astrocytes are small and electron lucent, with an estimated diameter of ~30 nm *in situ* [23] and ~50 nm when they recycle [36]. After Ca^2+^-dependent exocytosis [49–52], they are endocytosed [36]. Prior to entering into the endocytotic pathway, exocytotic vesicles may enter several rounds of recycling, where the transient exocytotic fusion pore reopens several times. Vesicles that transiently expose their lumen to the extracellular space may interact/uptake fluorescently labeled antibodies against VGLUT1. The antibodies were raised against amino acid residues thought to be present only in the cytoplasmic part of the VGLUT1 transporter protein. However, these residues are likely also present in the vesicle lumen in native vesicles, since anti-VGLUT1 antibodies label the luminal part of vesicles [36,53]. At higher intracellular concentrations of Ca^2+^ ([Ca^2+^]_i_) induced by 4 μM ionomycin or 1 mM ATP, the immunolabeling was more pronounced, and the directional mobility of VGLUT1 vesicles was increased. Together with directionality, TL, MD, and the fraction of fast-moving vesicles (>0.05 μM/s) increased at higher [Ca^2+^]_i_. These effects were absent in the cells preloaded with high affinity Ca^2+^ buffer BAPTA-AM. Microtubules, actin, and vimentin filaments likely play a role in the mobility process of VGLUT1 vesicles, because the disruption of actin attenuated their mobility [36]. As discussed by Stenovec *et al*. [36], regulation of VGLUT1 vesicle mobility after vesicle retrieval may likely be involved in various aspects of physiology, such as synaptic plasticity [54], silent synapses [55], astrocyte-to-neuron communication [3,56], and possibly more widely in cell biology, in the genesis and removal of vesicles from the plasma membrane [57].

### 2.2. ATP-Loaded Vesicles

ATP, an essential component of long-range calcium signaling in the nervous system [58], is also an important astrocytic gliotransmitter [13] and one of the major extracellular messengers for interastrocyte calcium-mediated communication [59,60]. In addition to nonvesicular modes of ATP release, such as the release of ATP from astrocytes mediated by the connexin hemichannel [61,62], volume-sensitive organic osmolyte and anion channels (VSOAC, [63]), Ca^2+^-dependent exocytotic ATP release from astrocytes has also been confirmed [13]. ATP-loaded astrocytic vesicles appear to be heterogeneous. So far, the vesicular distribution of ATP has been shown to overlap with the marker of dense-core granules in hippocampal astrocytes (SgII) [26,64] and in lysosomes [65–68], and it appears to be co- stored together with classic neurotransmitters (acetylcholine in neurons and noradrenaline in neurons and chromaffin cells [58]) or with peptides [22,26,64,69,70]. In neonatal cortical rat astrocytes, ATP-containing vesicles appear to be substantially co-stored with ANP (39 ± 7%) [22]. Under spontaneous conditions, the majority of ATP vesicles were located in close proximity to the plasma membrane (up to 150 nm) and this coincided with the observation that quinacrine-loaded vesicles displayed mainly nondirectional spontaneous mobility and only 4% of vesicles were highly mobile (directional mobility). High [Ca^2+^]_i_ affected both types of vesicle mobility and completely abolished directional movements. After triggered increase in [Ca^2+^]_i,_, fewer ATP vesicles were observed in the cells, likely due to the calcium-activated discharge of the fluorescent cargo by regulated exocytosis. This effect was obstructed by the presence of dominant-negative soluble NSF attachment protein receptor (SNARE) domain peptide, which interferes with the formation of the SNARE complex [22,49].

ATP is considered to be a major gliotransmitter in the propagation of calcium waves among astrocytes [3] and in the modulation of neuronal activity [71–73], the exocytotic release of ATP may play a role in the delivery of this gliotransmitter to the extracellular milieu as a signaling messenger for intercellular communication.

### 2.3. Peptide-Loaded Vesicles

Astrocytes store several peptides, including ANP, which is stored in membrane-bound vesicles [27]. The trafficking of ANP-loaded vesicles has been intensively studied in rat and mouse astrocytes, in cell cultures, and in tissue slices as a model of peptide vesicles. The extensive research regarding ANP vesicle mobility is presented as follows: the mobility in the secretory pathway was followed by the use of pro-ANP-Emd recombinant protein [74], and the mobility in the recycling pathway was monitored using an immunolabeling approach in living cells.

The major difference between the mobility of ANP vesicles monitored in the different pathways in rat astrocytes was the speed of the vesicles. The average speed of secretory vesicles (travelling from the cytoplasm to the plasma membrane) was 0.4 ± 0.007 μm/s (nondirectional vesicles 0.3 ± 0.005 μm/s, directional vesicles 0.5 ± 0.01 μm/s), indicating that all vesicles were mobile but some displayed straight line or directional motion. Such rapid vesicle mobility is comparable with the directional movement of vesicles in some neurons [19]. However, their mobility was significantly attenuated after depolymerization of microtubules, actin filaments, and intermediate filaments (IFs) to 0.30 ± 0.0003 μm/s, 0.08 ± 0.01 μm/s and 0.21 ± 0.01 μm/s, respectively [20]. In mouse astrocytes, the measured parameters of mobility were lower compared with rat astrocytes (Table 1). The slight difference in speed was also recorded between wild-type (WT) astrocytes and astrocytes without IF, and the extent of directional mobility was also lower in astrocytes without IF [20]. These data support the hypothesis that IFs are required for long-range directional vesicle mobility by acting as a three-dimensional lattice. The importance of astrocytic IF in vesicle mobility was also confirmed by more recent studies [21].

The mobility of ANP vesicles in the recycling pathway has been monitored in rat astrocytes. Vesicle recycling has been proposed for secretory granules, which are released by stimulated exocytosis [75]. During this process, the granule remains intact, except for the loss of the contents and some of the membrane proteins. Recycling occurs when the fusion pore is rapidly resealed in the exocytotic process and the vesicle is retrieved into the cytoplasm without intermixing of membranes and without collapse of the vesicle membrane into the surface membrane [75–77]. When studying recycling ANP vesicles, these exhibited one order of magnitude slower mobility than secretory ANP vesicles (Table 1). What is the physiologic significance of these results? The mobility of vesicles retrieved from the plasma membrane after exocytotic fusion is likely related to the efficiency of vesicle cargo discharge. If sufficient time is allowed (during attenuated vesicle mobility), the vesicle cargo can be completely discharged, especially if peptides in the vesicle are aggregated into dense matrices (detected as electron-dense material on electron microscopy) and their discharge is inefficient unless the vesicles exhibit a fusion pore that permits prolonged discharge of the vesicle cargo. Furthermore, brain ANP content is significantly increased after experimental brain infarction, but not in the brain haemorrhage, after contusion and in controls, indicating that ANP-positive astrocytes may increase in number, and may be involved in the regulation of the cerebral blood flow in the infarcted brain area [78]. The altered cerebral blood flow thus underlies also enhanced delivery of ANP vesicles to the plasma membrane and/or release of ANP from astrocytic vesicles.

In addition, a specialized form of bidirectional communication involving signaling peptides exists between neurons and astroglia. BDNF secreted from neurons in its precursor form (pro-BDNF) is cleared from the extracellular space into nearby astrocytes, which internalize it via formation of a complex with the pan-neurotrophin receptor p75 and subsequent clathrin-dependent endocytosis. Endocytosed pro-BDNF is then routed into a fast recycling pathway for subsequent SNARE-dependent secretion triggered by glutamate [40]. Similarly, tPA appears as an element of the crosstalk between neurons and astrocytes. tPA released by neurons is constitutively endocytosed by astrocytes via the low-density lipoprotein-related protein receptor, and is then exocytosed in a regulated manner. Here, however, the exocytotic recycling of tPA by astrocytes is inhibited by extracellular glutamate. Kainate receptors of astrocytes act as sensors of extracellular glutamate and, via a signaling pathway involving protein kinase C, modulate the exocytosis of tPA [41]. Apart from this, BDNF is the most prevalent growth factor in the central nervous system (CNS) and is widely implicated in psychiatric diseases, such as major depressive disorder (MDD), schizophrenia, addiction, and Rett syndrome [79]. Notably, *N*-methyl-d-aspartate receptor (NMDAR) antagonists may produce fast-acting behavioral antidepressant effects in depressed patients and studies in mouse models indicate that these effects depend on the rapid synthesis of BDNF. At cellular level, the blockade of NMDAR deactivates eukaryotic elongation factor 2 (eEF2) kinase (CaMKIII), resulting in reduced eEF2 phosphorylation and de-suppression of translation of BDNF [79]. Although the regulation of protein synthesis was identified as a valuable therapeutic target in treatment of MDD, one cannot rule out the possibility that fast-acting antidepressants affect trafficking and/or release of pre-synthesized BDNF from the brain cells; depressed patients report the alleviation of MDD symptoms within two hours of a single, low-dose intravenous infusion of the antidepressant drug [80].

Whether vesicles in intact tissue exhibit similar mobility to cultured cells was tested by labeling recycling vesicles in astrocytes in hippocampal tissue slices, because brain tissue slices represent a preparation that is physiologically closer to that occurring *in vivo*, *i.e.*, cell-to-cell contacts and tissue architecture are preserved as present in the brain [81]. We incubated brain slices from mice with antibodies either against ANP or against VGLUT1. Vesicles in astrocytes from the CA1 region of the hippocampus were recorded. The recording of vesicle mobility was performed with two-photon microscopy. The fluorescent puncta exhibited two types of mobility: nondirectional and directional. The average velocity of ANP-containing granules in slices from mice was approximately 0.04 μm/s, which is similar to that reported for recycling ANP granules in rat primary astrocyte cultures (0.06 μm/s) [31], but one order of magnitude slower than the velocity of prefusion pro-ANP-Emd labeled granules [19,20], where the average velocity was 0.4 μm/s. The mobility of VGLUT1 vesicles was analyzed similarly. The VGLUT1 vesicles in the slices were slightly slower than the ANP vesicles; their average velocity was approximately 0.03 μm/s. The velocity of recycling VGLUT1 vesicles in the slices was also slightly slower than recycling VGLUT1 vesicles from rat primary astrocyte cultures (0.05 μm/s) [36]. Although vesicle mobility may differ in different brain regions, these results show that the experimental data, which was obtained from cultured astrocytes, closely resemble the properties of vesicle mobility observed in tissue slices.

## 3. Endocytotic Vesicles

The mobility properties of endosomes/lysosomes have been described in detail in mouse astrocytes by Potokar *et al*. [32] and in rat astrocytes by Stenovec *et al*. [34]. These vesicles were labeled by LysoTracker dye (Ly) and exhibited slow mobility in comparison with other vesicle types (Table 1). The direction and speed of Ly vesicles was shown to be influenced by the absence of astrocyte IFs. The trafficking of Ly-labeled endosomes/lysosomes appears to be regulated differently from glutamate-containing (VGLUT1-positive) and peptide-containing (ANP-positive) vesicles under different physiologic conditions. Cell stimulation to trigger an increase in [Ca^2+^]_i_ significantly reduced the mobility of Ly-labeled vesicles in WT astrocytes but not in astrocytes devoid of IFs (*GFAP^−/−^Vim^−/−^* astrocytes) [82–84]. Moreover, stimulation-dependent regulation of VGLUT1- and ANP-positive vesicles was attenuated by the absence of IFs. Because these filaments get overexpressed under pathologic conditions [84], it is likely that vesicle traffic of distinct vesicle types is altered under these conditions [32], likely leading to vesicle traffic jams.

The regulation of endosome/lysosome mobility may exhibit completely different properties in pathophysiologic states. For example, if purified IgG antibodies harvested from patients with sporadic amyotrophic lateral sclerosis (ALS) are applied to astrocytes, the mobility of Ly-stained compartment(s) is transiently increased, likely in a calcium-dependent manner indicating that acidic compartments may not represent a functionally homogeneous subcellular compartment, although endosomes/lysosomes were stained predominantly [34]. How do these results relate to the disease? ALS is a complex, incurable, and non-cell autonomous degenerative disease that affects upper and lower motor neurons located in a neighborhood enriched with non-neuronal cells; its onset occurs in adulthood [85] with a projected lifetime risk of 1/2000 [86]. The hallmark of ALS is selective death of motor neurons, although glial cells are also affected. In ALS, astrocytic function is compromised in several ways that impair neuronal survival and includes: (1) deficient release of neurotrophic factors [87]; (2) release of nerve growth factor (NGF) or extracellular mutant superoxide dismutase 1 (SOD1) [88,89]; and (3) insufficient clearance of glutamate from the synaptic cleft, due to reduced density and loss of EAAT2 [90]. Disturbance of the physiologic balance between the neurons and astrocytes may therefore play a key role in motor neuron degeneration in ALS [91]. In addition, activation of a systemic immune response in patients with sALS [92] may play a role in the continuing pathology of ALS, once the blood–brain barrier is compromised [93]. Correspondingly, motor neurons survived less when cocultured on astrocytes expressing the mutant form of Cu-Zn SOD1, as in the familial type of ALS, than on WT astrocytes [94]. The application of conditioned medium from mutant SOD1-expressing astrocytes decreased the survival of motor neurons, suggesting the presence of astrocyte-secreting molecules that kill neurons [95]. Alterations in vesicle dynamics may thus reflect changes associated with the progression of the disease and may offer an expansion of available diagnostic tests.

## 4. Vesicles Transporting Aquaporins

The key molecule involved in brain water homeostasis is the AQP4, one of the three AQPs identified in brain cells *in vitro* and *in vivo* [96–100]. AQP4 isoforms in rodent and nonhuman primate brain are the most strongly expressed in astrocytic end feet surrounding the blood-brain barrier [99,101–103], and have also been identified in astrocytic processes in contact with synapses [99,104,105]. Several studies have suggested an important role of AQP4 in water transport in several physiologic processes including astrocyte swelling and brain edema formation/resolution under various pathologic conditions, both *in vitro* [106,107] and *in vivo* [10,108,109]. Water transport through the cell membrane may be regulated by the permeability properties of AQP4 [110,111], the heterogeneity of AQP4 crystalline-like orthogonal arrays of particles [112] and, as recently suggested, by the mobility of AQP4 vesicles to/from the plasma membrane [29]. AQP4e is one of the newly described basic AQP4 isoforms [113], and the properties of AQP4e vesicle mobility are described in a study by Potokar *et al*. [29]. In unstimulated conditions, the mobility of AQP4e vesicles resembled the mobility of slow recycling and endosomal vesicles [31,32,36] (Table 1). After dbcAMP treatment, a model to induce reactive astrocytosis, an increased AQP4 signal was measured at the plasma membrane after 15 min (and remained increased after 24 h) and the mobility of AQP4e vesicles was impaired: TL by 10% and MD by 15% [29]. These data indicate that the regulation of vesicle mobility in the short time scale is an important regulatory mechanism to alter the delivery/retraction ratio of AQP4 vesicles to/from the plasma membrane in reactive astrocytes. Decreased mobility with significantly lower directionality might contribute to restraining the AQP4 vesicles near the plasma membrane and may also be linked to dbcAMP-induced rearrangements of the F-actin cytoskeleton mesh already speculated to be one of the major factors responsible for increased AQP4 plasma membrane localization [114].

During the early stages of brain edema formation, astrocytes swell [11,12,109]. A reduction in osmolarity triggers an increase in soma volume; this has been measured in tissue and in cultured rat astrocytes [115–117]. The increase in cell volume may be accompanied by an increased rate of membrane insertion of exocytotic vesicles [118]. Potokar *et al*. [29] reported that hypoosmotic conditions affected plasma membrane localization of AQP4 in rat astrocytes, in particular hypoosmotic stimulation triggered a transient increase in AQP4 plasma membrane localization. These changes were related to changes in AQP4e vesicle traffic; an increase in AQP4 plasma membrane localization overlapped with the observed decrease in mobility of AQP4e vesicles and the subsequent decrease in AQP4 plasma membrane localization overlapped with increased AQP4e vesicle mobility. The changes in mobility occurred predominantly in directional vesicles.

## 5. Vesicles Delivering Plasma Membrane Transporters and Receptors

Intracellular traffic of astrocytic vesicles may also be utilized for the delivery of plasma membrane-associated receptors and transporters, such as MHC-II molecules [21,119] and glutamate transporter EAAT2 [120].

On exposure to proinflammatory cytokine interferon-γ (IFN-γ), otherwise immunologically silent astrocytes may begin to express MHC-II molecules and antigens on their surface and act as nonprofessional antigen-presenting cells (APCs). It has been suggested that IFN-γ-activated astrocytes participate in antigen presentation and activation of CD4 helper T cells in immune-mediated disorders of the CNS including multiple sclerosis [119,121] and experimental autoimmune encephalomyelitis [122].

In general, the delivery of MHC-II molecules from MHC-II compartments to the cell surface of APCs is mediated via a cytoskeletal network and is most likely completed with the fusion of MHC-II–carrying late endosomes/lysosomes with the plasma membrane. Actin microfilaments [123], microtubules [124,125] and their motor proteins [124,126] have been shown to mediate trafficking of MHC-II compartments in APCs. Only recently, the role of IFs in MHC-II trafficking was investigated in IFN-γ–activated astrocytes [21], which as reactive astrocytes overexpress IFs [127].

**Figure 3:**
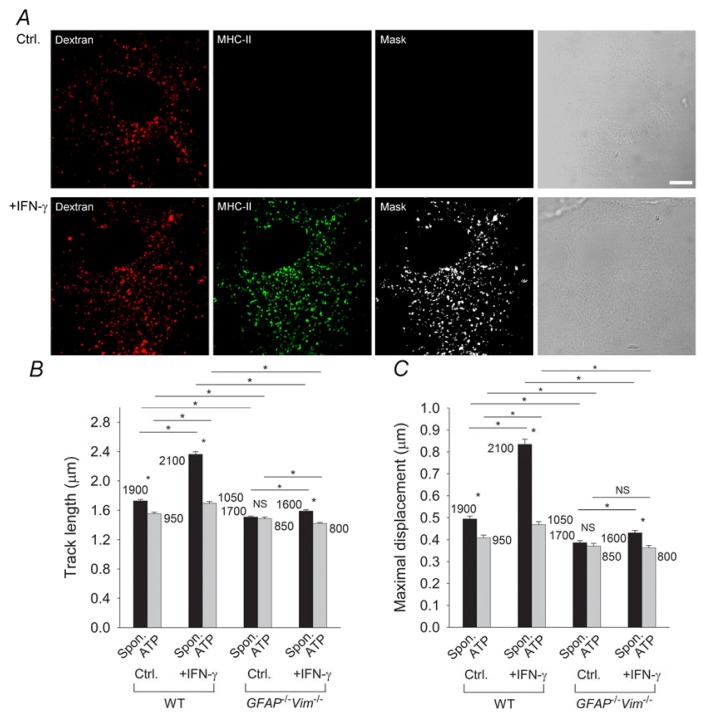
The IFN-γ-induced increase in the mobility of MHC-II compartments in astrocytes is IF dependent. (**A**) Alexa Fluor 546-dextran labels MHC-II–positive compartments in IFN-γ-treated WT and *GFAP^−/−^Vim^−/−^* (IF-deficient) primary mouse astrocytes. Fluorescence images of astrocytes labeled with dextran, fixed, and immunostained with antibodies against MHC-II molecules. White pixels (Mask) represent the colocalization mask of green (MHC-II) and red fluorescence pixels (Dextran). Scale bars: 10 μm; (**B**) Histogram of average vesicle track lengths in control (Ctrl.) and IFN-γ-treated (+IFN-γ) WT and *GFAP^−/−^Vim^−/−^* cells; (**C**) Histogram of the mean maximal displacements of vesicles in control (Ctrl.) and IFN-γ-treated (+IFN-γ) WT and *GFAP^−/−^Vim^−/−^* cells. Numbers on the bars are the numbers of vesicles analyzed. Values are mean ± SEM. ^*^p < 0.05. Adapted with permission from [21].

IFN-γ was shown to induce expression of MHC-II molecules on the astrocytic plasma membrane and late endosomes/lysosomes [21]. The latter could be specifically labeled with Alexa Fluor 546-conjugated dextran (Figure 3A) [21,128,129]. Time-lapse confocal imaging and dextran labeling of late endosomes/lysosomes in WT astrocytes and in astrocytes devoid of IFs (*GFAP^−/−^Vim^−/−^*) revealed faster and more directional movement of late endosomes/lysosomes in IFN-γ-treated astrocytes than in untreated astrocytes (Table 1). However, vesicle mobility was lower and less directional in IFN-γ-treated IF-deficient astrocytes than in WT astrocytes (Figure 3B,C), indicating that the IFN-γ-induced increase in the mobility of MHC-II-carrying late endosomes/lysosomes is IF dependent. Application of ATP and the subsequent increase in [Ca^2+^]_i_ induced attenuation of the mobility of late endosomes/lysosomes that was more apparent in the presence of IFs (Figure 3B,C), implying a role for IFs in this process.

These data indicate that, in IFN-γ-activated astrocytes, upregulation of IFs allows faster, and therefore more efficient, delivery of MHC-II molecules to the cell surface. Reduced mobility of late endosomes/lysosomes due to increase in [Ca^2+^]_i_ may increase their probability of docking and fusion [32], which, in astrocytes acting as APCs, may serve as an additional regulatory mechanism that controls the onset of late endosomal/lysosomal fusion and final delivery of MHC-II molecules to the cell surface [21]. Besides IFN-γ, endogenous suppressors, including norepinephrine, have been shown to regulate the expression of MHC-II molecules in astrocytes [130,131]. The effects of norepinephrine are mediated through activation of G-protein-coupled β-adrenergic receptors on astrocytes and subsequent activation of the cAMP signaling pathway. Our recent unpublished data suggest that the mobility and fusion of late endosomes/lysosomes involved in antigen presentation are also affected by activation of astrocytic β-adrenergic receptors. Although these studies were carried out *in vitro*, all these regulatory mechanisms may enable antigen-presenting reactive astrocytes *in vivo* to respond rapidly and in a controlled manner during CNS inflammation.

Astrocytes play a key role in the uptake of glutamate, which is released into the extracellular space from glutamatergic neurons during synaptic transmission [132,133] and from astrocytes themselves [37]. Physiologically, glutamate is cleared from the synaptic cleft via glial glutamate transporters GLAST (EAAT1) and GLT1 (EAAT2) [47]; its uptake is driven by the electrochemical gradient of sodium [134]. The flux of transported molecules also depends on the density of transporters in the cell plasma membrane [135,136], which determines whether synaptic independence is compromised by the synaptic transmitter crosstalk. The density of EAAT2 in astrocyte plasma membrane is regulated by exo-/endocytosis in a Ca^2+^-dependent manner [120]. Thus, the altered trafficking of EAAT2 to and from the plasma membrane may result in diminished net uptake of extracellular glutamate. An overabundance of glutamate accompanied by the failure of astrocytes to remove it, may lead to neuronal excitotoxicity resulting in a selective loss of motor neurons, as found in ALS.

## 6. Conclusions

Vesicle mobility studies on astrocytes have revealed that different types of vesicles exhibit specific properties. Moreover, during pathologic conditions, further changes may affect vesicle dynamics. This may affect the type of molecules that are released by vesicular mechanisms and/or may change the plasma membrane surface signaling landscape (altered densities of transporters, receptors, signaling mechanisms) that contributes to the intercellular communication between astrocytes and neighboring cells. The discovery that vesicle mobility may be modulated by pharmacological agents, such as Fingolimod/FTY720 [35], a recently introduced therapeutic for the treatment of multiple sclerosis [137], indicates that vesicle mobility may serve as a target for the development of new therapeutics for neurodegenerative diseases. It was shown that FTY720 accumulates in tissue hydrophobic pools [138], such as the white matter in the CNS, where it can reach concentrations that affect astrocytic vesicle mobility and consequently their ability to participate in regulated exocytosis [35], which may be part of its therapeutic effectiveness in patients with multiple sclerosis. Astrocytes were considered to be the major source of eicosanoids (prostaglandins, prostacyclins, thromboxanes, and leukotrienes), proinflammatory signaling molecules in the CNS that are released via an ATP-dependent mechanism [139]. Regulated exocytosis and vesicle traffic in astrocytes may thus represent a therapeutic target for a range of inflammatory states in the CNS.

## Figures and Tables

**Table 1:** Comparison of vesicle mobility properties in astrocytes.

Vesicle type (cargo)	Velocity (μm/s) (spontaneous)	Velocity (μm/s) (stimulation)	References	Comment *
Recycling (VGLUT1)	0.05 ± 0.02	0.13 ± 0.01 (ATP) 0.08 ± 0.00 (Iono)	Stenovec *et al*., 2007 [36]	Cell culture, rat
Recycling (VGLUT1) WT	0.06 ± 0.00	ND	Potokar *et al*., 2010 [32]	Cell culture, mouse
Recycling (VGLUT1) *GFAP^−/−^Vim^−/−^*	0.08 ± 0.00	ND	Potokar *et al*., 2010 [32]	Cell culture, mouse
Recycling (VGLUT1)	0.028 ± 0.001	ND	Potokar *et al*., 2009 [81]	Hippocampal slices, mouse
Recycling (ANP) WT	0.07 ± 0.00	ND	Potokar *et al*., 2010 [32]	Cell culture, mouse
Recycling (ANP) *GFAP^−/−^Vim^−/−^*	0.07 ± 0.00	ND	Potokar *et al*., 2010 [32]	Cell culture, mouse
Recycling (ANP)	0.037± 0.001	ND	Potokar *et al*., 2009 [81]	Hippocampal slices, mouse
Recycling (ANP)	0.06 ± 0.001	0.02 ± 0.002 (Iono), 0.03 ± 0.001 (ATP)	Potokar *et al*., 2008 [31]	Cell culture, rat
Secretory vesicle (ANP)	0.40 ± 0.007 0.29 ± 0.01	ND 0.27 ± 0.01 (Iono)	Potokar *et al*., 2005 [19]; Potokar *et al*., 2007 [20]	Cell culture, rat and mouse
Secretory vesicle (VGLUT1) Secretory vesicles (Sb2)	0.19 ± 0.02 0.65 ± 0.04	ND ND	Trkov *et al*., 2012 [35] Crippa *et al*.,2006 [42]	Cell culture, rat Cell culture, rat
Endo./lyso. WT	0.04 ± 0.00	ND	Potokar *et al*., 2010 [32]	Cell culture, mouse
Endo./lyso. *GFAP^−/−^Vim^−/−^*	0.04 ± 0.00	ND	Potokar *et al*., 2010 [32]	Cell culture, mouse
Late endo./lyso. WT	0.058 ± 0.001	0.052 ± 0.001 (ATP)	Vardjan *et al*., 2012 [21]	Cell culture, mouse
Late endo./lyso. *GFAP^−/−^Vim^−/−^*	0.050 ± 0.001	0.049 ± 0.001 (ATP)	Vardjan *et al*., 2012 [21]	Cell culture, mouse
Late endo./lyso. (MHC-II) WT	0.079 ± 0.001	0.057 ± 0.001 (ATP)	Vardjan *et al*., 2012 [21]	Cell culture, mouse
Late endo./lyso. (MHC-II*)* *GFAP^−/−^Vim^−/−^*	0.053 ± 0.001	0.047 ± 0.001 (ATP)	Vardjan *et al*., 2012 [21]	Cell culture, mouse
Late endo./lyso. (AQP4)	0.04 ± 0.00, 0.06 ± 0.00	ND	Potokar *et al*., 2013 [29]	Cell culture, rat
Endo./lyso. (CB1 receptor)	0.11	ND	Osborne *et al*., 2009 [14]	Cell culture, rat
Endo./lyso. (JAGGED/Notch)	0.06 ± 0.00	ND	Stenovec *et al*., unpublished	Cell culture, mouse
Endo./lyso.	0.11 ± 0.02	0.067±0.001 (Fingo)	Trkov *et al*., 2012 [35]	Cell culture, rat
Endo./lyso	0.21 ± 0.00	0.23 ± 0.00 (ALS IgG)	Stenovec *et al*., 2011 [34]	Cell culture, rat

Endo., endosome; lyso., lysosome; Iono, ionomycin; Fingo, fingolimod;

* Astrocyte cultures were prepared from the cerebrum of neonatal rats or mice.
